# Integrating PV-based energy production utilizing the existing infrastructure of MRT-6 at Dhaka, Bangladesh

**DOI:** 10.1016/j.heliyon.2024.e24078

**Published:** 2024-01-10

**Authors:** Md Ashraful Islam, Abdulla Al Mamun, M.M. Naushad Ali, Ratil H. Ashique, Abul Hasan, Md Majedul Hoque, Md Hasan Maruf, Md Ahmed Al Mansur, A.S.M. Shihavuddin

**Affiliations:** Department of EEE, Green University of Bangladesh, Dhaka, Bangladesh

**Keywords:** Renewable energy, PV plant, PV energy, Solar power, Metro rail, MRT line 6

## Abstract

In a very dense urban landscape, incorporating renewables becomes challenging due to a lack of space, planning, and mindset. Utilization of already existing large infrastructures in combination with existing technology and necessary adaptation can create the right synergy for harnessing renewables like solar. This paper proposes the installation of a solar power plant in Dhaka, Bangladesh, using available space on Metro Rail Line 6 to meet the increasing demand for clean and renewable energy. The proposed system involves the selection of suitable space, and PV panels, the calculation of annual electricity generation, and performing financial and environmental analyses. The proposed on-grid/grid-tied system offers the advantage of reducing dependence on power supplied to the grid, resulting in lower energy costs, and promoting the use of green energy. The system has a payback period of 7.7 years and a return on investment of 45.7 %. It is estimated that the system saves 14,053.203 tons of CO_2_ emissions per year and 281,064.06 tons of CO_2_ emissions over its lifetime. Also, the grid life cycle emission is 584 gCO_2_/kWh, and the system life cycle emission is 39,119.4 tCO_2_, which further proves that it is a feasible solution to meeting energy demands while reducing the dependency on fossil fuels and promoting sustainable energy utilization. The results of simulations run using PVsyst and HOMER confirm the economic viability of the proposed solar power station, supporting its viability. The levelized energy cost (LCOE), as projected by PVsyst, is $0.09 per kWh, nearly matching HOMER's prediction of $0.0835. This convergence of results from several simulation tools supports the solar power plant's predicted cost-effectiveness, demonstrating its potential as a key player in the effort to create a greener and more affordable energy landscape.

## Nomenclature

GDP: Gross Domestic Product
IPP: Independent Power Producer
JV: Joint Venture
NWPGCL: North-West Power Generation Company Ltd.
CMC: China National Machinery Import & Export Corporation
TCF: Trillion Cubic Feet
MRT: Mass Rapid Transit
URT: Urban Rail Transit
OCS: Overhead Catenary/Contact System
VDC, MAX: Maximum DC Voltage
VDC, Min: Minimum DC Voltage
PNOM, AC: Nominal AC Power
PMAX, AC: Maximum Output Power
VAC, NOM: Nominal AC Output Voltage
Voc: Open Circuit Voltage
Isc: Short Circuit Current
IAC, NOM: Nominal output AC current
MPPT: Maximum Power Point Tracking
DHI: Diffuse Horizontal Irradiation
LCOE: Levelized Cost of Energy

## 1 Introduction

Energy is essential for human survival and progress in today's world, and it also makes a major contribution to a country's security and economic development [1]. While much debate has been on the relationship between GDP and power use over the past year, their co-relational directions remain unclear. Numerous researchers have worked to address this issue and investigated the connection between electricity consumption and economic growth using data from both single countries and cross-countries over the past few decades. Earlier study results illustrate that electricity consumption can be taken as a crucial component for obtaining higher GDP growth in the short run [2,3]. Environmental pollution, clean energy, population density, urbanization, per capita GDP, and trade openness are also related [4,5]. Evidence of the relationship between GPD and electricity consumption can be found if one looks at the world's largest economies and their energy consumption [6,7]. Power demand has been rising each year along with global population growth, urbanization, and technological advancement [8,9].

Bangladesh is a developing country and has shown tremendously stable economic growth in recent times. According to the World Bank's report, Bangladesh is now a lower-middle-income country and is set to be a high-income country by 2041 [10]. Like other economically strong and developing countries, power demand in Bangladesh is also increasing rapidly. Per capita electricity generation capacity in the fiscal year 2022 was 470.02 kWh, while per capita consumption was 453.78 kWh in the fiscal year 2021 [11]. Bangladesh's power generation capacity as well as consumption have surely increased from before, but most of the power generation plants are based on fossil fuels [12,13]. In the fiscal year 2020–21, the electricity sector made considerable advancements in power production. The overall generation capacity reached 22,031 MW during the fiscal year after 2180 MW of additional generation capacity was installed, with an annual growth rate of 8.09 %. Out of this additional capacity, BPDB installed 1552 MW (including contractual capacity from IPPs), whereas NWPGCL added 6 MW and BCPCL installed 622 MW (JV of NWPGCL and CMC, China). The highest peak generation was 13,792 MW, and 80,423 GWh of energy was produced overall, which is an increase of 8.27 % and 12.61 % over the prior year, respectively [14]. In the fiscal years 2020–2021, domestic energy production accounted for 67.71 % of electricity production (60.19 % from natural gas, 6.21 % from coal, 0.81 % from hydropower, and 0.20 % from other renewable sources), 22.52 % from imported petroleum fuels (21.76 % from furnace oil and 0.76 % from diesel), and 10.08 % from imports. In the 2020–21 periods, domestic, industrial, commercial, agricultural, and other sectors utilized energy at rates of 56.42 %, 28.40 %, 10.58 %, 2.43 %, and 2.16 %, respectively [15]. But unsustainable energy systems impede Bangladesh's energy as well as economic growth. The primary challenge behind Bangladesh's development is its energy dependence on fossil fuels, which also has a significant impact on the achievement of the SDGs and Vision 2041. In addition, the Government of Bangladesh (GoB) is working with various private groups to meet the goal and is taking the initiative to produce electricity in the future from renewable sources, such as solar, wind, biogas, and biomass energy, among others. If energy security cannot be ensured, the vision of 2041 will not be feasible [[16], [17], [18]]. The data shows that Bangladesh produces the most electricity using fossil fuels and natural gas [19,20]. In general, fossil fuels are referred to as natural gas, coal, and petroleum [21,22]. Fossil fuels are often burned to produce electricity, and around 80 % of the world's energy comes from them, which releases a lot of greenhouse gases into the environment [23]. The total amount of recoverable natural gas was anticipated to be around 27 TCF by the end of 2017, and 15.22 TCF had already been consumed. Thus, 12 TCF of gas is still available, enough to satisfy the nation's needs for the subsequent 10–12 years only [24].

**Fig. 1:**
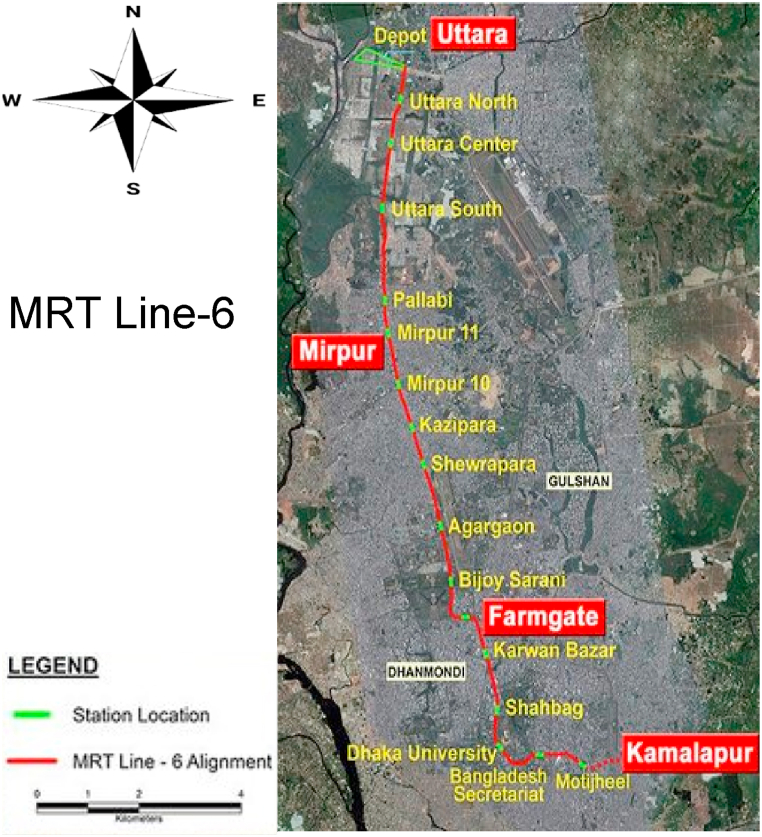
Route map of the MRT line 6 in Dhaka, Bangladesh. MRT Line 6 covers 21.26 km, with 17 stations from Uttara North to Motijheel.

Since Bangladesh's electricity is dependent on fossil fuels, it must be constantly imported to meet the electricity demand, which requires a lot of foreign exchange. Also, geopolitical, and unstable bilateral relations may increase fuel prices and affect Bangladesh's power generation, which hinders sustainable development. With over 169 million people, Bangladesh has ranked 8th on the list of countries by population [25], and Dhaka, the capital of Bangladesh, is the fourth megacity in the world in terms of population, where more than 22 million people live [26]. With so many people living in such a small space, there is a traffic jam due to the lack of space for movement. Studies have shown that it is ideal for a city to have 30–35 % area for roads [[27], [28], [29]], whereas Dhaka city has only 8 % for roads [30,31]**.** Due to a lack of roads and proper infrastructure, Dhaka suffers an unreasonable traffic jam daily. The transport sector contributes a huge amount of tailpipe emissions, and that's truer for Dhaka city. Dhaka City could reduce greenhouse gas emissions by utilizing its resources, mainly in the transport sector [32]. Metrorail is an important step of the Bangladesh government towards reducing this suffering on the roads in Dhaka city [32,33]. The work of MRT Line 6 from Uttara-Diabari in Dhaka to Kamalapur, shown in Fig. 1, which is 21.26 km long, is at the final stage [34]. There are 17 stations on this line, with the length of each station being 180 m. A few stations, including Diabari and Agargaon, are 30 m wide; a few stations, including Kazipara and Shawrapara, are 24 m wide; and the remaining stations are 27 m wide. The roofs of these stations are more than 23 m above the ground, and the rail tracks are about 13 m above the ground. These areas are mostly open and receive vast amounts of sunlight for most of the day [34]. This solar energy can be harnessed through solar panels to generate electricity, which will meet the electricity demand while also reducing dependence on fossil fuels.

X. J. Shen et al. check the feasibility and application modes of the PV generation system in Urban Rail Transit (URT) and get the results that the PV generation system is feasible at the typical line of Shanghai URT [35]. Flavio Ciccarelli et al. analysed the way PV panels can be integrated on a real tramway track and calculated PV energy [36]. The introduction of the first solar-powered railway track in the UK created new possibilities for the spread of this technology on railways all over the world. The rail track solar project was also established in Australia and India [37,38]. Binduhewa et al. discuss the limitations of integrating photovoltaic energy into a railway electrification system. It also presents five configurations that can be used to integrate photovoltaic into a railway electrification system, including integrating photovoltaic systems into high-voltage AC systems or medium-voltage AC railway electrification systems at the substation [39]. In Germany, the British company Bankset Energy is reportedly placing solar panels between the rails of Deutsche Bahn, or German Rail. This is known as a solar-mounted rail track. "A kilometre of railroad line may produce 100 kW of electricity [40]. Due to the global environmental problem, every industry must do its part to reduce emissions and slow down climate change. Every country is trying to utilize its available resources and increase the use of renewable energy [41,42]. Since to make sustainable development and meet the increasing energy demand, solar energy can provide the best possible solution. Solar energy is considered the greenest energy solution [43,44].

The objective of this paper is to propose the installation of a solar power plant in Dhaka, Bangladesh, using available space on Metro Rail Line 6 to meet the increasing demand for clean and renewable energy. The paper aims to select suitable space, PV panels, and calculate annual electricity generation, perform cost and environmental analyses, and propose a grid-tie structure that is connected to the electrical energy grid, allowing excess solar electricity to be transported for later use through net metering and clean energy credit creation. The paper also aims to estimate the system's payback period, return on investment, and CO_2_ emissions savings, and to demonstrate that the proposed solar power plant is a feasible and sustainable solution to meet the increasing demand for clean and renewable energy in Dhaka, Bangladesh.

The main contributions of this work could be summarized as follows.

1. In-depth analysis of PV panel integration at MRT Line 6 utilizing existing infrastructure and optimization of existing technologies
2. Cost analysis, impact assessment, and future projections reveal a payback period of 7.7 years and a reduction of 28,000 kg in carbon emissions.
3. Feasibility has been founded for the integration of this system in Dhaka, Bangladesh, considering weather conditions, energy demand, and government policies.
4. A total capacity of 22.11 MW can be installed, ensuring a levelized cost of energy (LCOE) of 0.09 dollars per kWh over the course of 20 years.

The paper's structure is organized as follows: Following the introduction, the Proposed PV Integration section provides an understanding of how photovoltaic integration is envisioned. In Suitable Space Allocation, we explore the potential use of unused space along MRT Line-6 for generating energy. The Grid Tie System Proposition explains why a grid-tie system is proposed. System Design offers a comprehensive system outline. Renewable Resources Allocation provides data on renewable resources in the study area. In the PVsyst Simulation and Result section, the outcomes from PVsyst software are presented. Economic and Financial Assessment discusses the economic and financial feasibility of the project. The HOMER Analysis section analyzes the project using HOMER optimization software. The Environment Analysis section outlines the environmental benefits of the study. Finally, the Conclusion section summarizes the study's findings concisely.

### 1.1 Proposed integration of PV

**Fig. 2:**
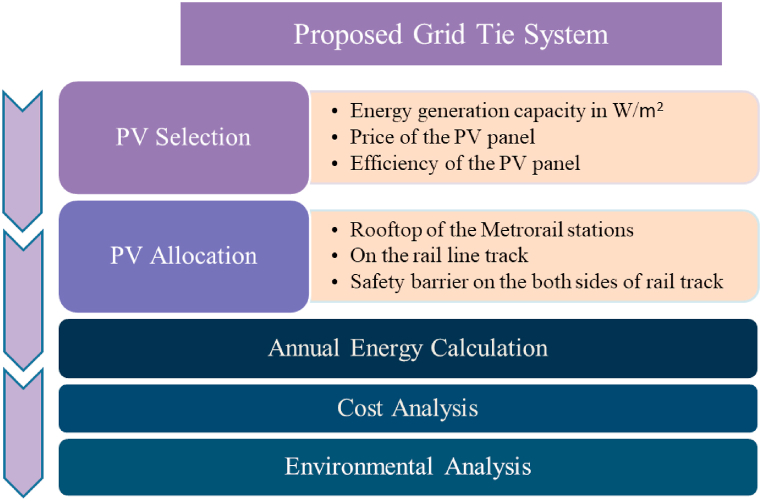
Step-by-step analysis to estimate the feasibility of the proposed PV installation on the MRT-6 Line infrastructure.

Metro Rail Transit (MRT) is a megaproject in Dhaka that needs huge investment and covers a huge area [32,45]. Solar power is becoming more popular as a feasible source of electricity because of the rising demand for clean, renewable energy. To meet the increasing energy demand in Dhaka, this paper proposes a solar power plant installed on the available space in the metro rail line, considering several factors such as sunlight intensity, useable area, and access to transmission infrastructure. Figure-2 depicts the detailed steps of the proposed system. This proposed solar energy power station in Dhaka using MRT Line 6 will be able to meet the increasing power demand, resulting in reducing the dependency on fossil fuels for electricity. The proposed system consists of.

• Space allocation in MRT line 6.
• Suitable PV panel selection.
• Annual electricity calculation
• Cost analysis and
• Environmental analysis.

**Fig. 3:**
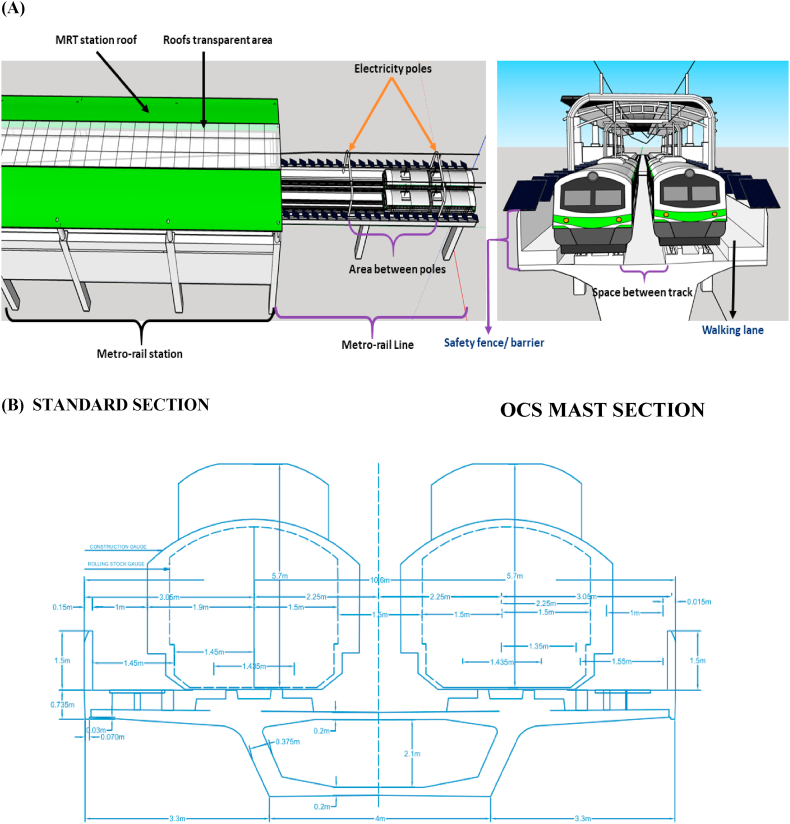
MRT Line 6 3D view is shown in A and cross-section view is shown in B. MRT-6 infrastructure's standard and OCS mast sections are also visible in the cross-section.

Outside the rail track and pole-mounted area, there is also a lot of unused available space (a safety fence or barrier between the pole and the roof of the MRT stations, see Figure-3A). Due to a lack of space and safety issues, solar panels cannot be installed between the rail lines. Suitable space allocation refers to the safe installation of solar panels without hampering the operation of the metro rail, which is analysed based on the specification and structure of the metro rail. Selecting the optimum size of the solar panels has been considered in this paper. In addition, the efficiency and price of the solar panels are presented along with their per-square-meter power generation capacity. The amount of annual electricity generation has been calculated using PVsyst and HOMER simulation software. A cost analysis has been done, which includes the levelized cost of energy and the payback period for the proposed system. It also determines whether the proposed system is feasible or not through simulation. The amount of CO_2_ reduction has also been calculated to justify the environmental analysis.

### 1.2 Suitable space allocation

Metro Rail Transit Line-6 (MRT-6) is a significant infrastructure project in Dhaka, Bangladesh, which spans an impressive 21.26 km and stands 13 m above the ground. The elevated position of MRT-6 makes it a safe and ideal location for installing solar panels, which can receive an ample amount of sunlight to generate renewable energy. Several countries around the world, such as Germany, Japan, India, and Australia, have already installed solar panels between conventional rail tracks to generate clean energy.

Figure-3B shows a cross-section view of MRT Line 6. However, MRT-6 is not constructed like a traditional rail track, and therefore, the empty spaces between the lines cannot accommodate solar panels. The width of MRT-6 is 10.6 m, including safety barriers and noise reduction walls, which are essential for protecting the safety of passengers and mitigating the noise level. The safety barrier has a height of 2.035 m and a width of 0.150 m. The distance between two sides of a rail line is 1.435 m, and the distance between two lines is 2.44 m.

In conventional rail lines, the stone is used to reduce vibrations during operation, while MRT-6 requires cast concrete for faster speed and frequency of travel. It is also helpful for an elevated position above the ground. Furthermore, to mitigate the issue of vibration and ensure smooth operation of the Metrorail line, cast concrete has been implemented with a vertical distance of only 75 mm between the train surface and the concrete. This distance is critical for addressing vibration and other factors that may arise during train operation. A 1-m distance is maintained between the train and the safety barrier when the train is on the line, ensuring the safety of the passengers. Due to the constraints imposed by the Metrorail line design, it is not feasible to install solar panels between the two paths of the rail lines. The ground-level space (2.44 m) indicated in Figure-3B is used for drainage, signal cabling, and maintenance of the lines and overhead electric cable. The two sides of the lines have a walking lane with a width of 1.45 m and 1.55 m, respectively, which are primarily utilized for maintenance and emergencies shown in Figure-3A. Although there are limitations to installing solar panels on MRT-6, including the limitation of space between the lines, there are still opportunities for mounting PV panels on the station rooftops and the safety barriers beside the tracks. These areas provide a suitable location for the installation of solar panels, considering the height of the Metrorail line and the ample amount of sunlight available at this elevation.

1. Station rooftop: The rooftops of Metrorail stations are each 180 m long, and the average width is 27 m. There is a transparent area of glass on the roof of the station only to receive light, except that there is a completely free space. There is 55,553 m^2^ of space available to use in all 17 stations after considering a 5 % space loss. If solar panels are installed in the space of the station roof, a lot of electricity can be generated which is shown in Table 1.

**Table 1:** Total available area calculation on all the station rooftop and line safety barriers in MRT-6.

The available area on the station rooftop	
Entry	Unit
Length of Station	180 m
The average width of the station	27 m
Average Width of the transparent area	7.79 m
The average area of a station	4860
The average area of transparent rooftop for daylight	1420.2
Area of all 17 stations without transparent rooftop	58,477
After considering the 5 % area loss, the total useable area on the station rooftop	55,553
**The available area on the line's safety barrier**	
**Entry**	**Unit**
The total length of the MRT line-6 without stations	18,200
10 % length loss for poles and other obstacles	32,770
Width of EVO6 pro panel	1.303
The total useable area on the line's safety barrier	42,588m^2^
Total useable area in MRT line-6 (Station rooftop and safety barrier)	98,151m^2^
2. Line and safety barrier: Barriers are constructed on both sides of the metro rail line for safety and to reduce noise pollution. On this barrier, solar panels can be installed without hampering metro rail operations. MRT line-6 is 18,200 m long excluding the length of the station. This study suggests installing PV panels on the safety barriers between electricity poles located on both sides of the metro-rail track. However, due to variations in the distance between these electricity poles, there will be unused spaces during the installation process. This is because the panel length and the space between poles are not uniform. So, a decent percent of space loss has been considered. After considering a 10 % length loss for the electric pole and other equipment, 16,380 m of space is available in each safety barrier with a total of 32,760 m area available for PV panel installation. The detailed calculation is shown in Table 1.

**Fig. 4:**
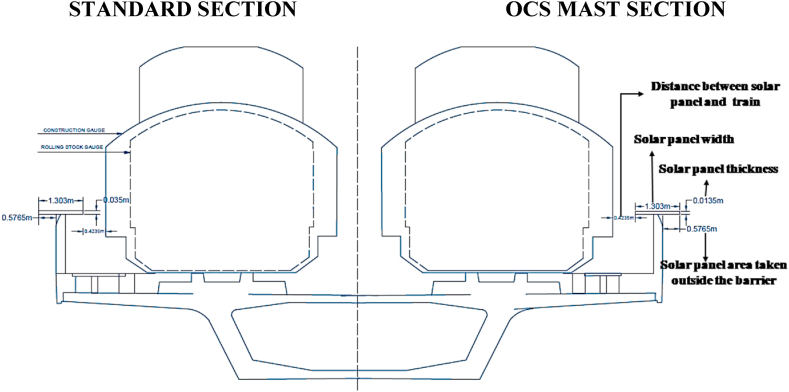
Cross-section view of MRT Line 6 with PV panel installation on safety barrier, panel dimensions: 1.303 m × 0.0135 m (Width* Length), requiring 0.58 m on each side of the safety wall. The train will be 0.42 m from the safety barrier fence solar panel.

**Fig. 5:**
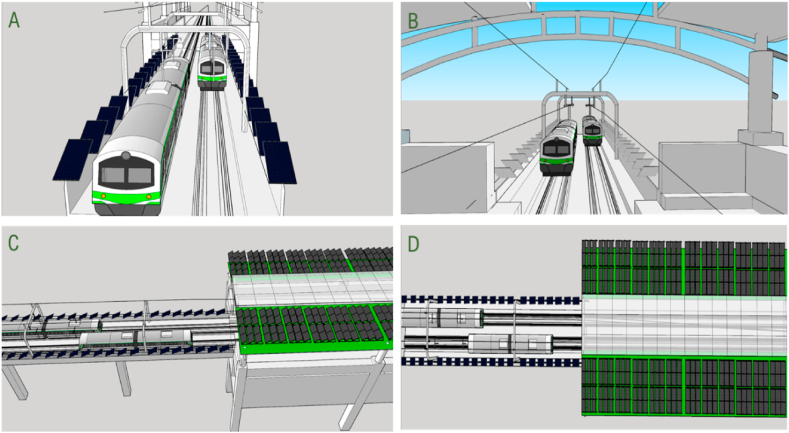
SketchUp 3D model of proposed PV allocation: Subsection A - Front view of MRT-6 with PV panel on safety barrier. Subsection B - Back-side view of MRT Line-6 with PV panel. Subsection C - Side view and half of the rooftop. Sub-figure D - Top view of the rooftop.

Fig. 4 shows how PV panels can be installed on the safety wall on both sides of the Metrorail track, ensuring enough safety. Fig. 5 shows the 3D view of PV installed on the Metrorail station rooftop and the safety barrier.

**Grid Tie System:** The implementation of solar panel installation on Metro Rail Line 6 in Dhaka City is proposed in this study due to the scarcity of available space and the vast area covered by the Metrorail project. The proposed grid-tied solar device is connected to the electrical energy grid, allowing for the transportation of excess solar electricity for later use, net metering, and the creation of clean energy credits. Grid-connected photovoltaic systems are a type of renewable energy source that is integrated into the electrical grid. One of the most significant advantages of a grid-tie solar energy system is its cost-effectiveness when compared to off-grid solar systems. Unlike off-grid systems, grid-tied solar devices do not require battery storage to store energy, as they directly deliver the energy from sunlight into the grid. The grid-tie inverter is a crucial component of the system that converts DC energy to AC and feeds it into the grid connection, reducing dependence on purchased energy and promoting the use of green energy. By reducing the reliance on power supplied from the grid, the proposed system would result in a lower cost of energy consumption. The use of solar energy offers an abundant source of renewable energy that can be utilized to not only save electricity costs but also promote the use of green energy. The proposed on-grid/grid tie system offers a cost-effective and environmentally friendly solution that would not only benefit the Metrorail project but also contribute to the city's sustainable development. Therefore, this paper advocates for the implementation of the proposed system to harness the potential of solar energy and promote sustainable development in Dhaka City.

### 1.3 System design

A review of some available PV model information on the market is shown in Table 2. This paper follows some key guides on choosing a solar panel described in the System Design section to consider the most suitable panel among all kinds of available solar panels on the market. These are possibly the first and most important factors for considering a suitable solar panel when comparing panels.

**Table 2:** Comparison list of several types of PV models and their efficiency, price, and watts per square meter.

PV Model	Efficiency (%)	Price $ USD	W/m^2^
MAXEON 6 AC [46]	22.8	505.5	227.7
EVO6 Pro [47]	22.5	186.9	225.4
HiHero CS6R-H-AG [48]	22.5	215.3	225.3
Alpha Pure R [49]	22.3	252.7	222.8

The evaluation in Table-2 shows that the EVO 6 Pro model's solar panel offers better output for less cost. The efficiency of this panel is 22.5℅ and the price of each panel is USD 186.9. MAXEON 6 AC PV [46] panel has the highest efficiency of 22.8 % currently available on the market but its price is much higher than other panels compared to the price per watt ratio. EVO 6 Pro and HiHero CS6R-H-AG PV panels have the same efficiency but it takes slightly more space to produce the same amount of power and the price is more than selected PV EVO 6 Pro. Information about selected PV is shown in Table-3 and all the information is collected from the verified website.

**Table 3:** All the technical information of the selected PV module for EVO6 Pro [47].

Parameters	Value/Units
Model	SE6-66HBD
Manufacturer	SunEvo Solar
Panel dimension (L*W*H^1^)	2384 x 1303 × 35 mm
Panel weight	38.2 kg
Cell type	Silicon wafers
Number of cells	132 Cells
Nominal power	700W
Maximum Voltage	42.10V
Open circuit voltage, Voc	50.13V
Short circuit current, Isc	19.22A
Panel efficiency	22.5 %
Degradation Rate	0.6 %
Warranty	30 Years

Inverter: Table-4 outlines crucial details of the selected inverter, FreeSun FS0880 HES 330V, highlighting key parameters and features. Manufactured by Power Electronics, this inverter boasts a maximum input voltage of 1000 VDC, a Multi MPPT feature for efficient power tracking, and a nominal AC power of 800 kWac. The table provides information on input and output specifications, including voltage, current, and efficiency, contributing to an insightful overview of the inverter's capabilities and characteristics.

**Table 4:** Selected inverter information for FreeSun FS0880 HES 330 [50].

Parameters	Value/Unit
Manufacturer	Power Electronics
Model	FreeSun FS0880 HES 330V
Max. input voltage, **V**_**DC, MAX**_	1000
Min, input voltage, **V**_**DC, Min**_	430
MPP voltage range, **V**_**DC**_	430
MAX, input current, **I**_**DC**_, _**max**_	1920
Number of DC input	32
Nominal AC power, _**PNOM, AC**_	800kWac
Max. output power, _**PMAX, AC**_	800
Nominal output AC current _**IAC, NOM**_	1711
Nominal AC output voltage _**VAC, NOM**_	270
Max. efficiency	98.55 %
Frequency	50
Weight	4,500 kg
MPPT feature	Multi MPPT

**Energy storage system:** A major disadvantage of energy storage systems for solar power plants is their cost. Energy storage systems, such as batteries, are expensive to purchase and need to tolerate hassle while maintaining, which can increase the overall cost of the solar power plant [60]. Another drawback of the energy storage battery system is the short lifetime of the battery. Additionally, the energy storage systems may not be able to store all the energy generated by the solar power plant, which could result in wasted energy. Another disadvantage is the environmental impact of the energy storage system, as some batteries use materials that can be toxic and harmful to the environment [51].

### 1.4 Monthly solar irradiation and position of the sun in Dhaka

**Fig. 6:**
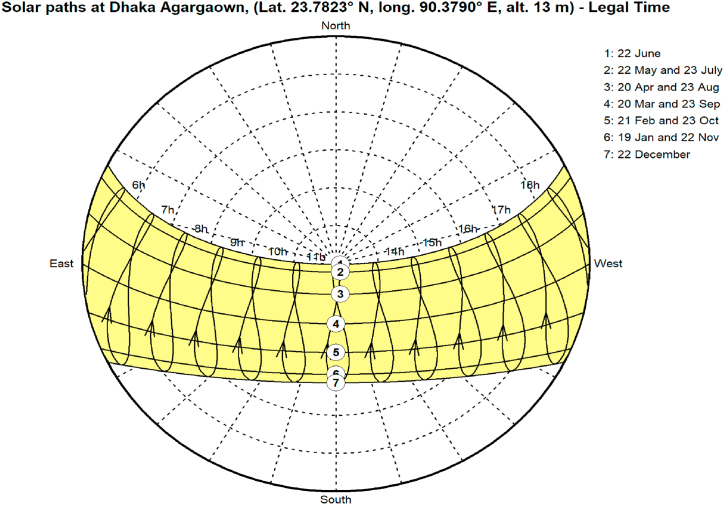
Daily sun paths for Dhaka city: Illustrates the sun's path throughout the day and year, aiding solar energy planning and building design that optimize sun exposure.

**Fig. 7:**
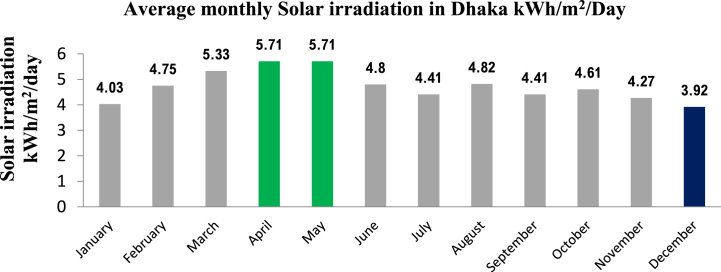
Monthly solar irradiation in Dhaka city. The highest solar irradiation in Dhaka city is 5.71 kWh/m^2^/day in April and May and the lowest solar irradiation is 3.92 kWh/m^2^/day.

Due to its subtropical location in South Asia, Bangladesh receives abundant solar radiation throughout the year. Several factors, such as geographic location and the sun's angle, which fluctuates from 40 to 90° as shown in Fig. 6, contribute to this. The average daily solar radiation in Bangladesh varies from 235 W/m^2^ in the south-to-west to 215 W/m^2^ in the north-to-west. Dhaka, the capital city of Bangladesh, is situated in the country's central region and receives high levels of solar radiation throughout the year. The average monthly solar irradiation in Dhaka is shown in Fig. 7. Like the rest of Bangladesh, Dhaka experiences an average daily solar radiation of 4–6 kWh/m^2^/day. The highest sun radiation levels of 5.71 kWh/m^2^/day are observed during April and May, while the lowest sun radiation levels of 3.92 kWh/m^2^/day are observed in December. Based on geographical standing, the yearly direct nominal irradiance (DNI) in the Metrorail area is roughly 988.6 kWh/m^2^, and the diffuse horizontal irradiation (DHI) is around 949.1 kWh/m^2^ [52]. However, factors such as urbanization and tall buildings in the city can affect solar radiation levels. These factors may cause some solar radiation to be partially reflected or obstructed, which can reduce the amount of solar energy that is useable in the area. Despite this, the city still receives a substantial amount of solar radiation, making it a favourable location for installing solar energy systems.

## 2 PVsyst analysis

### 2.1 PVsyst simulation parameters

PVsyst is a software tool widely used for simulating the performance of grid-connected photovoltaic (PV) systems. The software can be used to design, analyse, and optimize a solar power plant's performance by considering various parameters such as weather data, solar panel information, system configurations, orientation, the tilt angle of the PV panel, and different types of losses [52,53]. In this context, understanding the different parameters that impact the performance of a PV system is crucial to developing an efficient and cost-effective solar power plant. Therefore, this section will discuss the PVsyst simulation parameters used in this study, including the data set, load data, and system configuration.

**Data Set:** For the development of a grid-connected photovoltaic system, it's crucial to gather vital data that impacts the system's performance. The two data sets that have a significant impact on system constraints are geographical data and load data. To prevent oversizing or under-sizing the system, load data calculation is essential in grid-connected solar systems, which drives up the system's cost.

**Tilt angle:** It is important to find and use the optimum tilt angle for the selected site to get the most possible output from the designed system. To calculate the optimum tilt for solar panels, the following sum can be used:

90° – your latitude = optimum fixed year-round setting [54].

For the fixed-axis system, the optimum tilt angle for Dhaka city is 23°, and the azimuth angle is 0°, which means a south-facing panel [55].

**Fig. 8:**
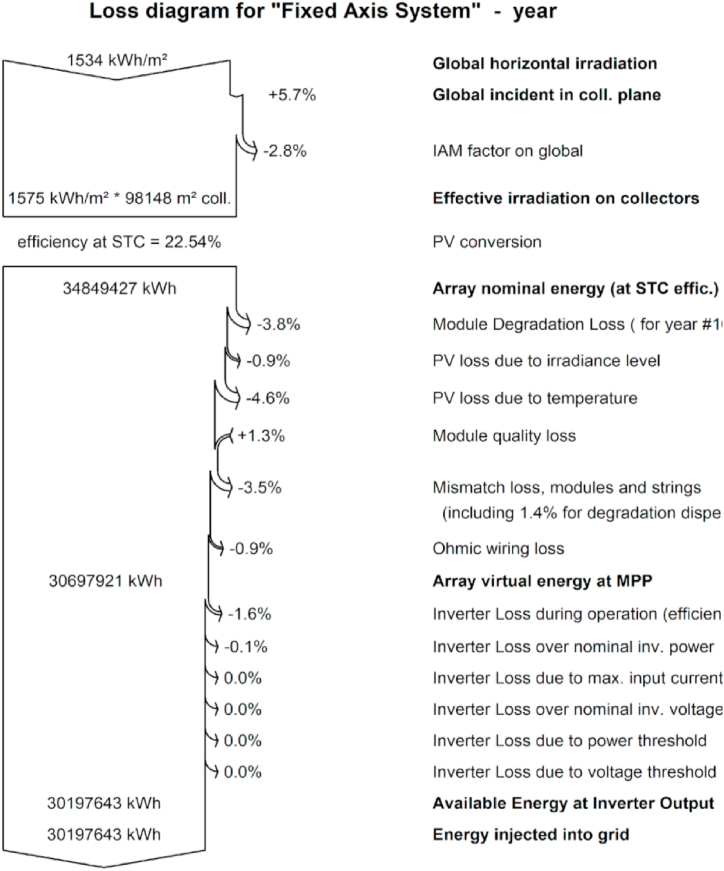
Loss diagram for fixed axis grid tie solar tracking system.

**Detailed Losses:** A solar power plant may experience a variety of losses, which could impair the system's functionality as an entire. Electrical losses, thermal losses, and mechanical losses are the three basic categories into which these losses can be divided. Fig. 8 shows the losses of the fixed-axis grid-tied solar tracking system [56].

## 3 Simulation and results

### 3.1 Incident irradiation distribution

**Fig. 9:**
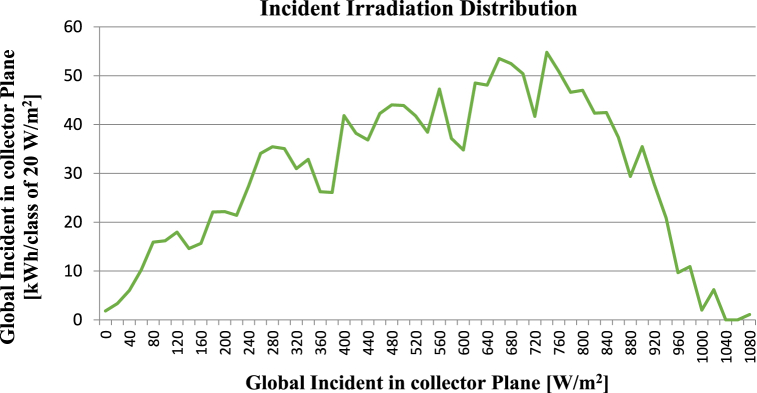
Incident Irradiation Distribution graph: Illustrates global incident irradiation distribution, with X-axis in watts per square meter and Y-axis in kilowatt-hours per class of 20 W per square meter.

The Incident Irradiation Distribution graph presents important insights into a solar system's feasibility. This graph can be examined to identify the amount and distribution of solar radiation that is present at a particular place. This data is essential to figure out the solar power plant's potential output and whether the location is suitable for a PV system. The x-axis of the graph represents the quantity of incident solar energy that the solar panel surface has received over time. The frequency distribution of the irradiation data is displayed on the y-axis. The Incident Irradiation Graph in Fig. 9 for this study shows a consistent and high level of irradiation throughout the day, which indicates that the site is suitable for a solar power plant as it receives a significant amount of sunlight.

**Fig. 10:**
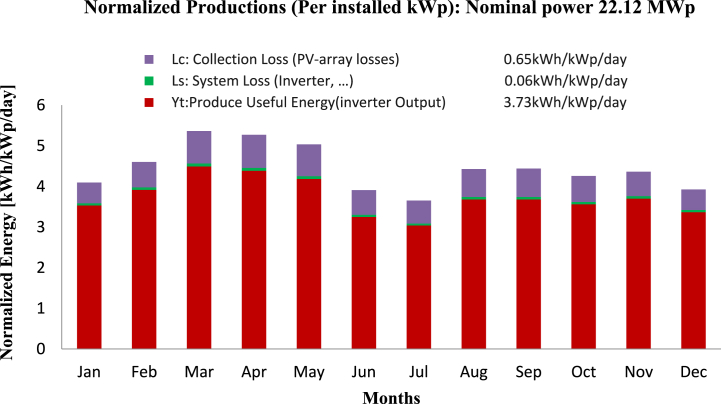
Normalized Production for 22.12 MW Solar Plant: Monthly performance graph, includes inverter output (3.73 kWh/kWp/day), system loss (0.06 kWh/kWp/day), and collection loss (0.65 kWh/kWp/day).

**Normalized Productions (Per installed kWp):** The normalized production (per installed kWp) graph illustrates how much energy is produced overall by the solar panel system as an entire unit. This graph provides a visual representation of the monthly variation in the solar power plant's performance and the contribution of each component to the normalized energy output. An assessment of the system's overall viability can be made by looking at this graph. The Figure-10 graph for this simulation shows a consistent and stable production output per installed kWp, which suggests that the system is performing efficiently and as expected. This gives confidence that the system is feasible and can deliver the expected energy output.

### 3.2 Normalized production and loss factors

**Fig. 11:**
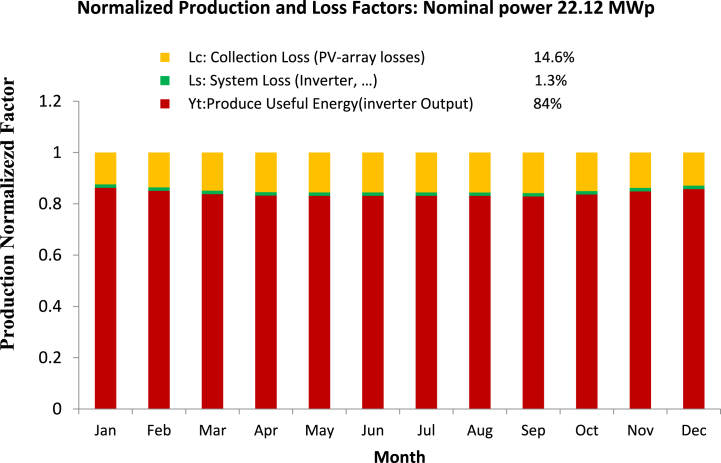
Normalized Production & Loss Factors: 22.12 MW Solar Plant. Graph shows monthly production factor considering inverter output, system loss (1.3 %), collection loss (14.6 %), and useful energy (84 %).

The Normalized Production and Loss Factors graph provides valuable insights into the efficiency and performance of a photovoltaic system, as shown in Fig. 11. The graph displays the percentage of energy produced under different weather conditions and the contributing factors to energy loss. An analysis of the graph identifies the factors causing energy loss, such as shading, soiling, and temperature losses. These insights guide decisions on system design, maintenance, and cleaning schedules to optimize the system's performance and minimize energy loss. Based on the results of this study simulation, the normalized production and loss factors graph for the nominal power 22.12 MWp system shows a 16 % loss. This value is within an acceptable range for the MRT project, as it falls within the industry-standard range of 10–20 % for large-scale solar systems.

**Fig. 12:**
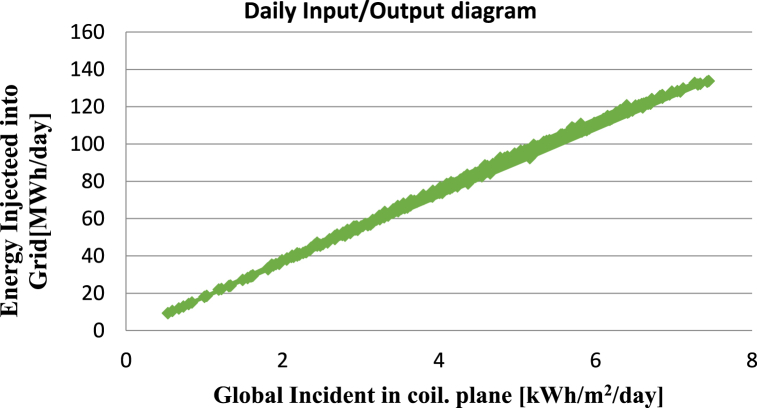
Daily energy input/output diagram for proposed solar power system.

**Daily input/output diagram:** The relationship between the worldwide incidence in the collector plane and the energy injected into the grid for a hypothetical solar power system is shown in the Daily Energy Input and Output Diagram. The graph can be used to determine whether the system is providing the load with enough energy and whether there are any periods of surplus or deficit energy. The system's configuration and design may be improved with the aid of this data. For instance, it is possible to alter the system's component sizes and use different types of energy storage. The graph also offers perceptions of the system's general effectiveness and performance, enabling an assessment of any potential improvement areas. The Daily input/output diagram for this study simulation in Figure-12 shows that a constant increase is an indicator that the system is performing well and consistently meeting its energy production targets.

**Fig. 13:**
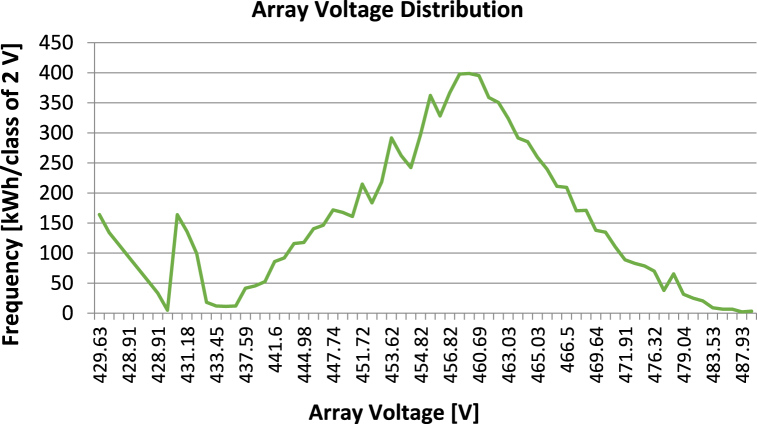
Array voltage distribution for proposed solar power system.

**Array Voltage Distribution:** The Array Voltage Distribution graph provides details about the voltage distribution across the solar panel array. This graph can be used to identify any hotspots or low-voltage regions that may exist in the array. These hotspots or low-voltage regions might result from soiling, shadowing, or other conditions that lower the solar panel array's efficiency. The Array Voltage Distribution graph for this study simulation in Figure-13 shows a uniform voltage distribution across the array, with no hotspots or low voltage areas. This indicates that the array is working efficiently and that there are no significant issues that need to be addressed.

**Fig. 14:**
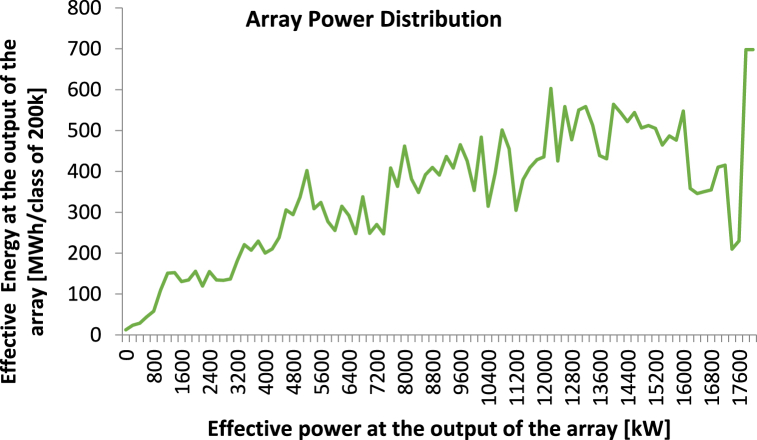
Array Power Distribution for proposed Solar Power System. The x-axis represents the effective power at the output of the array in kW, while the y-axis shows the effective energy at the output of the array in MWh per class of 200 kW.

**Array Power Distribution:** Array power distribution graph shows the power output of each string or module in a photovoltaic system. By analysing this graph, it is possible to identify any potential issues in the system, such as shading, module mismatch, or wiring issues, which may cause a reduction in power output. Simulation in the Figure-14 graph shows a balanced and consistent distribution of power and energy output across the solar array. This indicates that the system is working efficiently and effectively to convert solar energy into useable power.

**Fig. 15:**
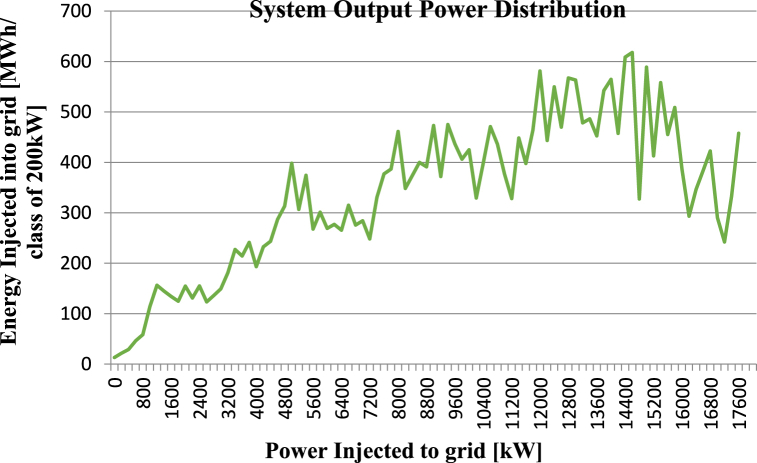
System Output Power Distribution for Proposed Solar Power System. The x-axis shows the power injected into the grid in kW, while the y-axis displays the energy injected into the grid in MWh per class of 200 kW.

**System Output Power Distribution:** The graph represents the distribution of energy output from the proposed solar power system. By analysing the graph, it is possible to identify the periods when the system produces the maximum and minimum energy output. This information can be used to make decisions regarding energy storage requirements, grid connection, and system design. The graph in Figure-15 shows a consistent and high energy output during the peak demand period, which indicates the necessity of connecting the system to the grid to sell the excess energy produced during this period.

**Fig. 16:**
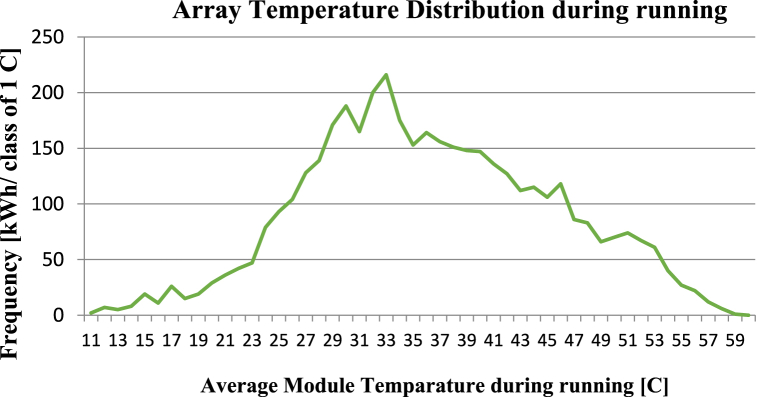
Temperature Distribution of Solar Array during Operation. The x-axis represents the temperature in degrees Celsius, while the y-axis represents the frequency in kWh per class of 1 °C.

**Array Temperature Distribution during running:** The Array Temperature Distribution during running provides information about the temperature distribution of solar modules during system operation. By analysing the graph, it is possible to identify areas of the array that are overheating, which can cause a decrease in system efficiency and lead to long-term damage to the modules. The simulation graph in Figure-16 has a temperature distribution that is relatively uniform across the array, indicating that the modules are operating efficiently and are not experiencing excessive losses due to temperature.

**Fig. 17:**
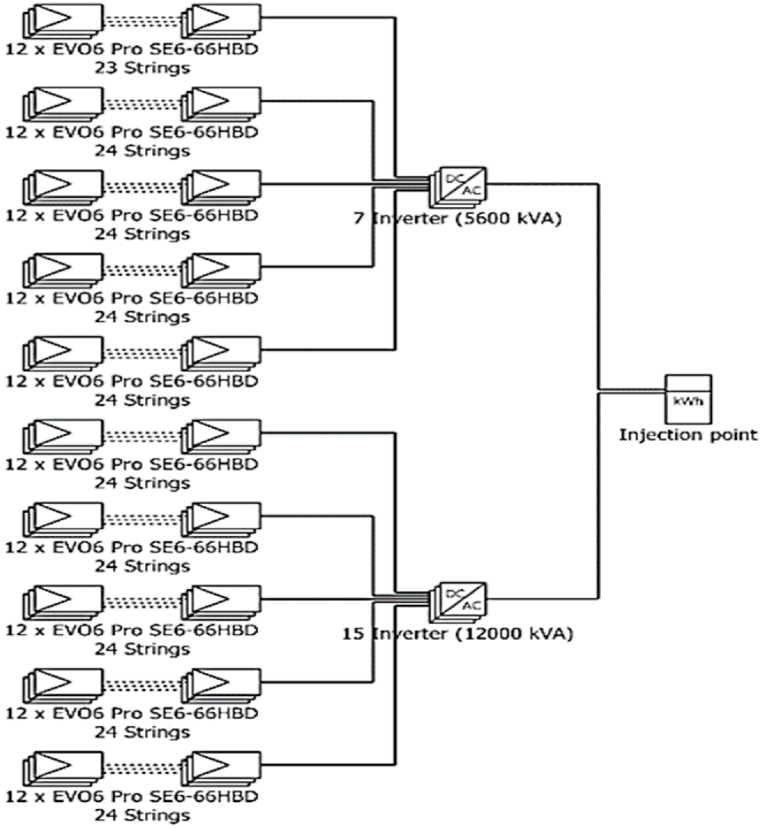
Single-line diagram for the proposed on-grid solar power project.

**Single line Diagram for the proposed project:** The single-line diagram obtained from PVsyst simulation for the proposed solar power system shown in Figure-17 comprises a total of 7 strings, consisting of 12 EVO6 Pro 24 modules each and one 2EVO6 Pro 23 string. Out of the ten strings, nine are 12*EVO6 Pro 24 strings, and another is 12*EVO6 Pro 23. Among these five 12*EVO6 Pro 24 strings are connected to a total of 15 inverters. The remaining strings are connected to seven inverters. The power generated from these inverters is transmitted to the injection point through two lines, which are then fed into the grid. This single-line diagram provides a comprehensive overview of the electrical components and their connections in the solar power system, which is essential for ensuring the safe and efficient operation of the system.

The Technical specifications and performance parameters of a proposed photovoltaic (PV) system using MRT Line-6 are shown in Table-5. The proposed PV system has a nominal DC power of 22,117 kWp and a maximum DC power of 22,006 kWDC. The nominal AC power of the system is 17,600 kWAC with a Pnom ratio of 1.26. The maximum voltage at the operating condition is 645V, and the maximum current is 45.89 KA. The system consists of 31,596 PV modules, divided into 2633 strings with 12 modules in each string, covering an area of 98,148 m2. The system has 22 inverters and 44 MPPT inputs. The specific production of the proposed PV system is 1361 kWh/kWp/year, and the produced energy without loss is 30.11 GWh/year. This table's data can be used to make informed decisions about system design, installation, and maintenance, as well as to assess the viability and performance of the suggested PV system.

**Table 5:** Specifications and performance parameters of the 22.1 MWp solar PV power plant

Specification	Value
Nominal PV Power, _PNOM, DC_	22,117 kWp
Maximum PV Power, _PMAX, DC_	22,006 kWDC
Nominal AC Power, _PNOM, AC_	17,600 kWAC
P_nom_ Ratio	1.26
Maximum Voltage at Operating Condition	645V
Maximum Current at Operating Condition	45.89 KA
No. of PV Modules	31,596
No. of Strings	2633
No. of PV Modules in Each String	12
Module Area	98,148 m^2^
No. of Inverters	22
No. of MPPT Inputs	44
Specific Production	1361 kWh/kWp/year
Produced Energy (Without Loss)	30.11 GWh/year

**Economical Assessment:** An economic assessment of a solar power plant is a process of evaluating the financial viability of the project. It involves analysing the costs and benefits of the project over a specific period, typically the lifetime of the plant. The initial capital costs of a solar power plant, such as the cost of the solar panels, inverters, mounting systems, and other equipment, as well as the costs of site acquisition, permits, and installation, are included in the costs of a solar power plant. The prices also cover any potential repairs or replacements, as well as continuing maintenance charges such as the price of cleaning and maintaining the solar panels. Table-6 shows the cost estimation of this photovoltaic (PV) system installation, including the cost of PV modules, inverters, other components, studies and analysis, installation, and maintenance. The total cost of the project is around $9 million, and the estimated operational expenditure (OPEX) is around $1.3 million. The table also includes provisions for inverter replacement, salaries, repairs, security funds, and insurance. The estimated depreciable asset value is around $8 million.

**Table 6:** The initial capital cost of this power plant including each piece of equipment as well as mounting systems, installation, and depreciable assets.

Sl no	Purpose	Items	Cost $(USD)
1	PV modules	EVO6 Pro SE6-66HBD	5,905,292
2	Inverters	FreeSun FS0800 HE/HEC 270V	2,200,000
3	Other components	Wiring	300,000
		Combiner box	131,500
		PV module structure	1579
		Energy meters	850
		PV testing equipment	2000
4	Studies and analysis	Survey and inspection	50,000
		Engineering drawing	50,000
		Environmental studies	10,000
		Economic analysis	10,000
5	Installation	Installation cost per module	315,960
		Installation cost per inverter	1100
		Transport	10,000
		Grid connection	10,000
**Total**			8,998,282
**Depreciable asset**			8,105,292
**Operating costs**			
6	Maintenance	Provision for inverter replacement	220,000
		Salaries	500,000
		Repairs	10,000
		Security fund	500,000
		Insurance	50,000
**Total (OPEX)**			1,280,000
**Total (OPEX including inflation)**			2,765,410

**Financial Assessment:** Financial assessment for this solar power plant involves evaluating the costs and potential revenues associated with this project. This covers the price of the hardware and installation, ongoing maintenance expenses, and possible earnings from selling the electricity the solar panels produce. These assessments also consider factors such as the cost of financing and any government incentives or subsidies available for the project. Table-7 provides information on the economic parameters of this MRT project. This project has a lifetime of 20 years, and its total installation cost is around 9 million USD, of which the depreciable asset is around 8 million USD. The funding for the project is a loan with an interest rate of 1.25 % per year. The operating cost (OPEX) is around 2.7 million USD per year, and the loan annuities are 0.5 million USD per year. The net present value of the project is around 4.1 million USD. The feed-in tariff for the electricity produced by the project is 0.10 USD/kWh, and the levelized cost of energy (LCOE) is 0.0879 USD/kWh. The payback period for the project is 7.7 years, and the return on investment (ROI) is 45.7 % for this project.

**Table 7:** The table shows the total cost of the project, project lifetime also other factor costs, and returns on investment. As well as comparing the cost and revenue.

Parameter	Value (USD)
Project Lifetime	20 years
Total Installation Cost	8,998,282
Depreciable Asset	8,105,292
Funding	Loan
Interest Rate	1.25 % [International rate from the world bank] 2
Operating Costs (OPEX)	2,765,410 USD/year
Loan Annuities	498,642.64 USD/year
Net Present Value	4,115,095
Feed-In Tariff	0.10 USD/kWh
Levelized Cost of Energy (LCOE)	**0.0879 USD/kWh**
Payback Period	7.7 years
Return On Investment	45.7 %

**Fig. 18:**
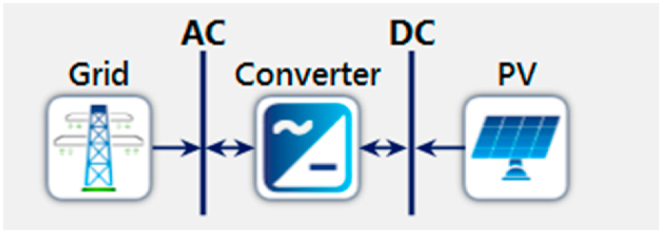
System configuration of on-grid system.

**HOMER Analysis:** This HOMER analysis examines optimization results, technical specifications, and cost information for grid-tie systems. Fig. 18 presents the system configuration of a PV and grid-connected system generated from HOMER simulation software.

**Fig. 19:**
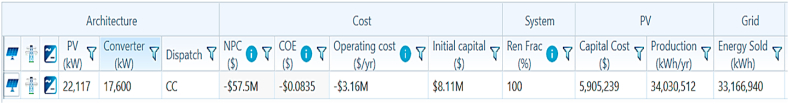
HOMER optimization result.

**Fig. 20:**
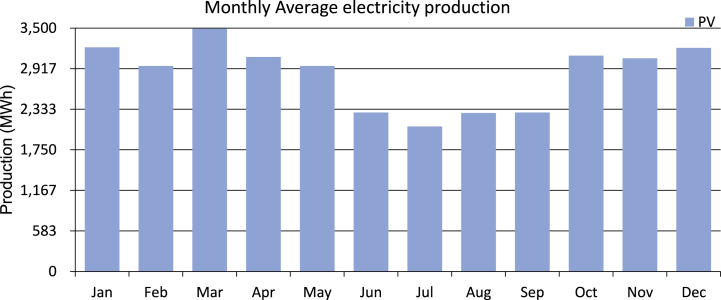
Monthly average electricity production.

Fig. 19 shows the system-optimized result by HOMER. The HOMER calculated Net Present Cost for the system is $57.5 M, the annual operating cost is $3.16 M, and the system requires an initial capital cost of $8.11 M. HOMER shows annual system production is 34.03 GWh, and the amount of energy sold is 33.17 GWh after avoiding the loss. The levelized cost of energy for this system by HOMER is $0.0835 per kWh, which is almost the same as the PVsyst analysis. Fig. 20 shows monthly average electricity production.

Table-8 shows the per-unit costs and lifetime CO_2_ emission reductions of various solar power plants operational in Bangladesh, along with the location and capacity [[57], [58], [59]]. Comparing these figures to the LCOE of the MRT Line-6 project, which is 0.0879 USD/kWh with a CO_2_ emission reduction of 281 k tons during its system life, the MRT Line-6 project has a significantly lower LCOE compared to the other solar power plants in the table. Lower LCOE results in more economically efficient energy generation, which is a major benefit for the MRT Line-6 project. The project's ability to eliminate 281 k tons of CO_2_ emissions over the course of its system life is also a crucial advantage, helping to provide a more ecologically friendly and sustainable energy solution.

**Table 8:** Compared between several of the solar park name, capacities, and locations, furthermore also their cost list in BD and USD

Sl No	Solar Park name	Capacity (MW_P_)	Location	Cost/kWh ($)	Lifetime CO_2_ Reduction (Kilo ton)
1	Sympa Solar Power ltd.	8	Panchagarh	0.117	82
2	Engreen Solar	3	Sarishabari, Jamalpur	0.171	31
3	Technaf Solartech Energy ltd.	20	Teknaf, Coxs Bazar	0.125	206
4	Spectra Solar Park	35	Shibalaya, Manikganj	0.110	361
5	Energon Renewables (BD) ltd.	100	Mongla, Bagerhat	0.117	100 M ton
6	Sirajganj 6.13 MW Solar Photovoltaic Power Plant	7.6	Sirajganj Sadar	0.14	78
7	HDFC Sin Power ltd.	50	Gauripur, Mymensigh	0.153	516

**Environmental Assessment:** The Environmental Assessment (EA) for this solar power plant study evaluates the potential environmental impacts of the proposed project. Outcome of environmental assessment is shown in Table-9. The total system life cycle emission is found to be 39119.4 tCO_2_ for this project, which indicates the amount of carbon dioxide emitted during the manufacturing, installation, operation, and disposal of the system. The grid life cycle emission is estimated to be 584 gCO_2_/kWh, which is the amount of carbon dioxide emitted per unit of electricity produced by the grid. The installation of the PV system has resulted in significant environmental benefits, as it has saved 14053.2 tCO_2_/year compared to the conventional grid. This amount of CO_2_ emission reduction corresponds to 281064.06 tons over the expected 20-year lifetime of the PV system. Therefore, the installation of the PV system has contributed to mitigating climate change by reducing greenhouse gas emissions.

**Table 9:** Various parameter values of environmental analysis consisting of the period of this proposed power plant.

Parameters	Value
Grid Life Cycle Emission	584 gCO_2_/kWh
System Life Cycle Emission	39119.4 tCO_2_
Saved CO_2_ Emissions per year	14053.2 tCO_2_/year
Saved CO_2_ Emissions	**281064.06 Tons**

**Discussion:** Metro Rail Transit (MRT) is a megaproject in Dhaka that needs huge capital costs and covers a massive area. There are a lot of unused areas inside metro rail infrastructure that can be used to install PV panels as a renewable energy generation source. This study tries to check the feasibility of renewable energy generation using PV panels at MRT Line-6 stations and line tracks. The study shows that in MRT line 6 there are 98,151 m^2^ available areas in which PV panels can be installed, including the roof of the MRT station and the safety fence of the railway line, which are proposed for the installation of solar panels. A strict process has been followed for selecting compatible solar panels and inverters. The selected PV panel for this project is EVO6 Pro manufactured by SunEvo Solar, and the selected inverter is FreeSun FS0880 HES 330, manufactured by Power Electronics Company. To minimize the project cost and avoid maintenance hassles, a grid tied system has been proposed. In the time of system design, the amount of solar irradiation and the sun path of Dhaka city have been considered. Electrical, thermal, and mechanical losses have also been considered. Table-10 summarizes the key findings of the proposed system. Our proposed system has the lowest Levelized Cost of Electricity (LCOE) of $0.09 per kW compared to other available solar plants in Table 8. Additionally, the system's ability to reduce CO2 emissions by 14,053 tons per year is another important factor to consider. It's worth noting that the payback period for this system is 7.7 years.

**Table 10:** Key findings of the proposed PV system

Parameters	Value
Capacity	22.11 MW
Payback Period	7.7 years
LCOE	0.09 $/kW
Lifetime CO_2_ reduction	281064.06 Tons

**Conclusion:** To address the growing need for clean and renewable energy, the paper suggests building a solar power plant in Dhaka, Bangladesh, using space on Metro Rail Line 6. The suggested system entails choosing an appropriate location, installing PV panels, calculating the amount of electricity generated annually, and doing economic and environmental evaluations. The system is based on a grid-tie structure that is linked to the electrical energy grid and enables the transportation of extra solar electricity for later use through net metering and the development of clean energy credits. Reduced reliance on power supplied to the grid, cheaper energy costs, and encouragement of the use of green energy are all benefits of the proposed on-grid/grid-tie system. The system has a return on investment of 45.7 % and a payback period of 7.7 years. The system is predicted to reduce CO_2_ emissions by 281,064.06 tons over the course of its lifetime and 14,053.203 tons annually. The system life cycle emission is 39,119.4 Ton-CO_2_, and the grid life cycle emission is 584 gCO_2_/kWh, demonstrating that it is a practical way to meet energy needs while encouraging sustainable energy sources and reducing reliance on fossil fuels. Future studies should concentrate on improving the suggested system, looking into energy storage options, and assessing how such initiatives might affect the broader energy infrastructure. The suggested solar power plant, though, is peculiar to the context of Dhaka, Bangladesh, and could not be applicable to other places with different climatic and economic conditions. Additionally, this article has not considered the various difficulties in implementing the suggested system, such as legal and policy obstacles, technical problems, and social acceptance. The projected solar power plant is, all things considered, a promising way to meet the rising need for clean, renewable energy and it has the potential to help create a more sustainable future. In order to lessen the effects of climate change and build a more resilient and sustainable world for future generations, it is our collective responsibility to accept and support such projects.

## Data availability statement

The data supporting the findings of this study can be obtained from the corresponding author upon reasonable request. Specific details regarding space allocation, technology selection, as well as measurements encompassing the length, height, cross-section, and number of stations pertaining to MRT-6 are available and can be referenced in the publication's Reference 35.

## Additional information

No additional information is available for this paper.

## CRediT authorship contribution statement

**Md Ashraful Islam:** Writing – original draft, Methodology, Formal analysis, Conceptualization. **Abdulla Al Mamun:** Writing – review & editing, Methodology, Investigation, Formal analysis. **M.M. Naushad Ali:** Writing – review & editing, Writing – original draft, Supervision, Formal analysis. **Ratil H. Ashique:** Writing – review & editing, Resources, Conceptualization. **Abul Hasan:** Software, Formal analysis, Data curation. **Md Majedul Hoque:** Resources, Methodology, Investigation. **Md Hasan Maruf:** Investigation, Formal analysis, Conceptualization. **Md Ahmed Al Mansur:** Writing – review & editing, Supervision, Formal analysis. **A.S.M. Shihavuddin:** Writing – review & editing, Supervision, Formal analysis, Data curation.

## Declaration of competing interest

The authors declare that they have no known competing financial interests or personal relationships that could have appeared to influence the work reported in this paper.

## Footnotes

1 Solar panel dimensions are expressed as L*W*H. Height of the solar panel is referred to as the thickness of the solar panel in this paper.

2 https://www.worldbank.org/en/news/press-release/2019/03/01/world-bank-helps-bangladesh-expand-renewable-energy.
